# A concave four-arc honeycomb with enhanced stiffness and desirable negative Poisson’s effect

**DOI:** 10.1038/s41598-023-48570-y

**Published:** 2023-11-30

**Authors:** Ning Feng, Yuanhao Tie, Ronghui Guo, Qingwen Yuan, Fengling Xue, Cheng Li, Liwen Lv, Weibo Xie

**Affiliations:** 1 School of Intelligent Manufacturing and Transportation, Chongqing Vocational Institute of Engineering, Chongqing, 402260 China; 2 Henan Province Engineering Research Center of Ultrasonic Technology Application, Pingdingshan University, Pingdingshan, 467000 China; 3 College of Mechanical and Electrical Engineering, Xinjiang Agricultural University, Urumqi, 830052 China; 4 School of Mechanical and Automotive Engineering, Guangxi University of Science and Technology, Liuzhou, 545006 China; 5 School of Resources and Security, Chongqing Vocational Institute of Engineering, Chongqing, 402260 China

**Keywords:** Mechanical properties, Mechanical engineering

## Abstract

The conventional star-shaped honeycomb (CSSH) structure is inherently rich in mechanical properties. Based on the CSSH structure, the Poisson's ratio and Young’s modulus can be improved by adding the tip re-entrant angle (ISSH). In this paper, a new concave four-arc honeycomb (CFAH) structure is proposed by designing the straight rod as a curved rod and retaining the tip re-entrant angle from the ISSH structure. The Young's modulus, specific stiffness and Poisson’s ratio of CFAH structures are derived from Castigliano’s second theorem and Moore’s theorem. The theoretical results show good agreement with the numerical and experimental results. The results show that the normalized effective specific stiffness and normalized effective Young’s modulus of the CFAH structure are further improved by about 12.95% and 16.86%, respectively, compared with the ISSH structure, and more significant auxiliary effects are obtained. CFAH structures show good promise in aerospace, construction and other applications due to their enhanced mechanical property. Meanwhile, the present work provides guidance for the study of concave four-arc honeycomb structures.

## Introduction

In the previous decades, metamaterial^1,2^ structures have attracted the attention and research of many scholars due to their excellent properties^3,4^. Artificially designed metamaterials have properties that natural materials do not possess^5^. These properties arise from the internal macrostructure rather than the constituent materials, and they can often be precisely programmed by changing the geometry and parameters of the cells. These properties include strong impact resistance^6^, high indentation resistance^7^, lightweight^8^, high strength^9^, high stiffness^10^ and excellent energy absorption properties^11^. Due to their diverse properties, metamaterials are used in many fields such as civil engineering^12^, aerospace engineering^13^, mechanical engineering^14^, vehicle engineering^15^, acoustics^16^, optics^17^, etc. The most classical of the extraordinary properties of metamaterials are the negative Poisson's ratio effect^18–20^ and the zero Poisson's ratio effect^21–23^. Compared with conventional honeycomb structures, honeycomb structures with negative Poisson's ratio effect have more excellent mechanical properties and show good prospects for applications in the automotive and aerospace fields. NPR materials shrink laterally when compressed along the longitudinal direction^24^, rather than expanding^25^. They usually have good shear modulus^26^, enhanced bending strength^27^, and excellent energy absorption properties. However, the ZPR effect is the opposite of the NPR effect^21^. When the ZPR material is compressed, its lateral dimensions remain in their initial state and do not shrink or expand^28^.

In 1987, Lakes^29^ made the first artificial polyurethane foam with NPR. Subsequently, various honeycomb structures have been discovered by researchers^30,31^, such as re-entrant structure^32–34^, chiral structure^35^, arrow-head^36^ structure and star structure^37,38^. Meanwhile, with the rapid development of additive manufacturing technology, the study of honeycomb structures has become more intensive. Feng et al.^39^ investigated the in-plane mechanical properties of annular honeycomb structures by experimental, theoretical and finite element methods. The results showed that the Poisson’s ratio of the annular honeycomb structure can be transformed from positive to zero Poisson’s ratio by changing the geometric parameters of the structure. Baran et al.^40^ enhanced the conventional folded honeycomb by adding new inclined walls. This was investigated by experimental, theoretical and FE methods. The results showed that the in-plane stiffness of the folded back cells was enhanced by this design. Choudhry et al.^41^ investigated the in-plane energy absorption characteristics of a conventional re-entrant assisted honeycomb by improving its design with finite element and experimental methods. The results showed that the modified structure had an increased damage strain and a 36% increase in specific energy absorption capacity. Qiang et al.^42^ investigated the impact resistance of auxiliary Double arrowhead honeycombs (DAHs) structures by theoretical and finite element methods. While the results showed that the quasi-static yield stress of DAHs depends on the geometrical parameters and NPR, the deformation mechanism related to the impact resistance of DAHs is discussed. Liu et al.^43^ studied the star-shaped honeycomb structure with different tip folding-in angles and proposed multiple improved star-shaped honeycombs with adjustable Poisson's ratio. The mechanical characteristics of the three-dimensional star-shaped honeycomb were also studied based on the two-dimensional star-shaped honeycomb. The results showed that the 3D honeycomb exhibited higher strength and stability when subjected to compressive loads. Zhang et al.^44^ studied a novel two-dimensional arc-shaped star structure and its three-dimensional configuration. The effective Poisson's ratio and effective Young's modulus of the structure were investigated numerically as well as by using the energy method. The results showed that the effective Poisson's ratio of the structure can be programmed from positive to negative by changing the geometric coefficients.

In previous studies, the conventional star-shaped honeycomb (CSSH) structure has received extensive attention and research. With the in-depth study, researchers have designed and improved the CSSH, and many novel star-shaped honeycomb structures with excellent performance have emerged. The improved star-shaped honeycomb (ISSH) structure is designed to replace the tip angle by adding a re-entrant angle, and this design improves the mechanical properties of the structure. The ISSH structure is mainly composed of straight rods, and the mechanical properties after changing the straight rods to curved rods are unknown. The purpose of this paper is to fill this gap and to provide a more comprehensive and accurate guide for the design of star-shaped honeycomb structures. In this paper, a concave four-arc honeycomb (CFAH) structure is proposed, which replaces the straight rod of the ISSH honeycomb with a curved rod. The CFAH structure was investigated by combining theoretical, finite element (FE) and experimental methods. Interestingly, we find that the normalized effective Young's modulus and normalized effective specific stiffness of the structure are greatly improved after designing the straight rod as a curved rod, and show more auxiliary effects. The present work takes the study of star-shaped honeycomb structures a step further.

## Structural evolution

**Figure 1:**
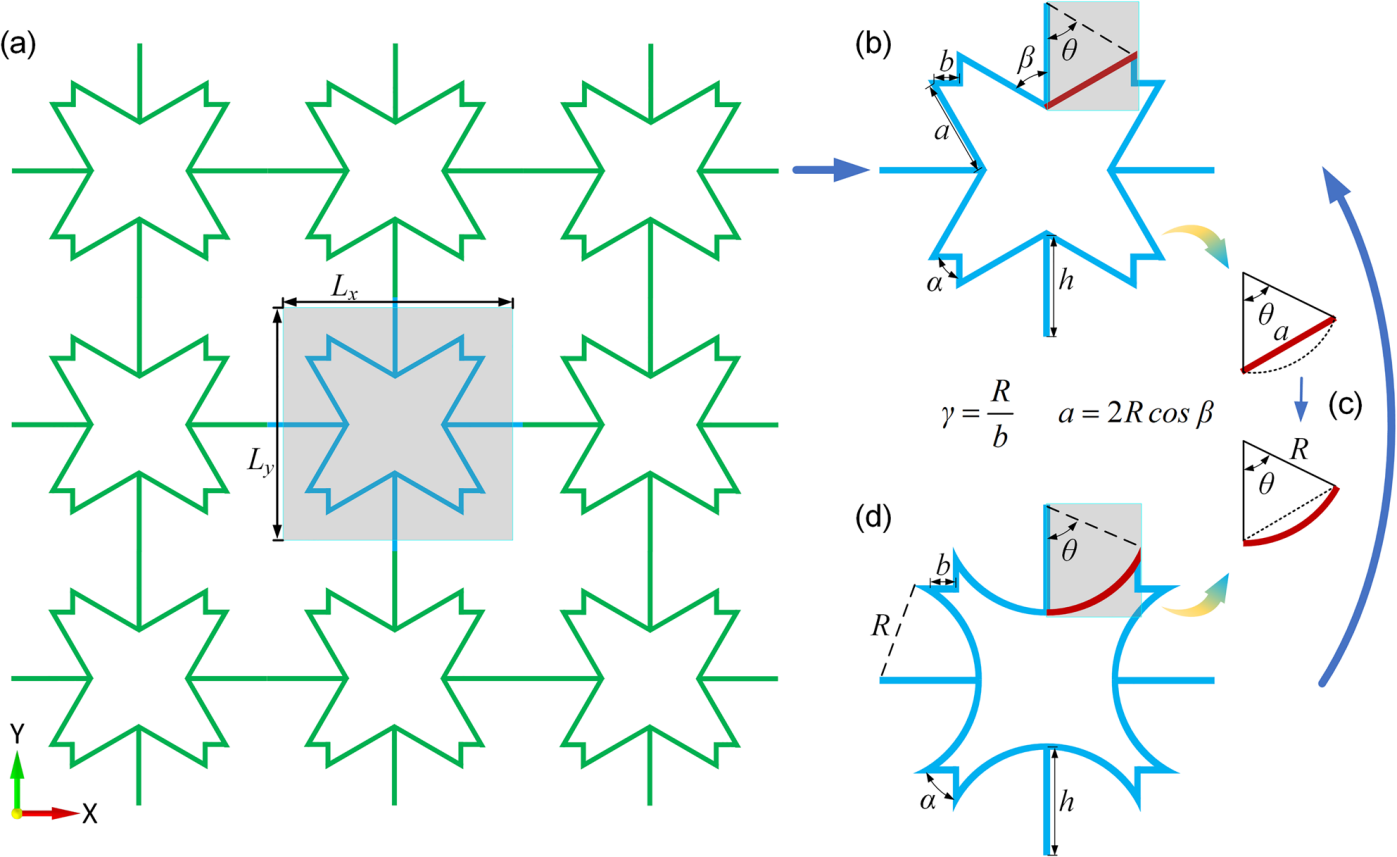
(**a**) ISSH array rules; (**b**) ISSH unit cell^43^; (**c**) the change process; (**d**) CFAH unit cell.

The improved star-shaped honeycomb (ISSH) structure is described by folding the tip angle of the conventional star-shaped honeycomb (CSSH) structure inward, as shown in Fig. 1a. Compared with the ISSH structure, the geometry of the concave four-arc honeycomb (CFAH) structure is designed with the inclined rod as a curved rod, as shown in Fig. 1c. Figure 1b and d show the differences between the two structures at the inclined rods. The geometry of the ISSH structure can be described by seven parameters, including *a*, *b*, *β*, *θ*, *α*, *h*, and *t*. The geometry of the CFAH structure can be described by six parameters, including *R*, *b*, *β*, *θ*, *α*, *h*, *t*. The parameter *b* is the length of the short rod, the parameter *h* is the ligament length, and the parameter *α* (*α* = 90°) is the tip re-entrant angle. The unit cell length and width are the same and are denoted by *L*_*x*_ and *L*_*y*_, respectively. To simplify the calculation, a dimensionless parameter *γ* is introduced, which is defined as the ratio of *R* to *b*. The length of the inclined rod is described by the parameter *R*.

1 $$\gamma = \frac{R}{b}$$

2 $$a = 2R{\text{cos}}\beta$$

### Relative density

The relative density of a porous material is defined as the ratio of the density of the structural units to the density of the structural material. It is an important parameter that affects the mechanical properties of the structure. The relative densities of the ISSH and CFAH structures are calculated analytically as follows:

3 $$\rho_{s}^{ISSH} = \frac{{4t\left( {h + 2a + 2b} \right)}}{{L_{x} L_{y} }}$$

4 $$\rho_{s}^{CFAH} = \frac{{4t\left( {h + 2b + 2R\theta } \right)}}{{L_{x} L_{y} }}$$

## Results and discussions

In this section, the effects of structural parameters on the elastic properties of CFAH structures are first discussed. The differences in Poisson's ratio, normalized Young's modulus and normalized specific stiffness between the ISSH and CFAH structures are then compared. Strikingly, the material required to fabricate the CFAH structure is slightly higher than that of the ISSH structure, but the normalized effective Young’s modulus and normalized effective specific stiffness increase by about 16.86% and 12.95%, respectively. Meanwhile, the negative Poisson effect is more significant. These results demonstrate the superior performance of the CFAH structure in terms of cost-effectiveness and application potential.

### Geometric description and theoretical analysis of elastic properties

**Figure 2:**
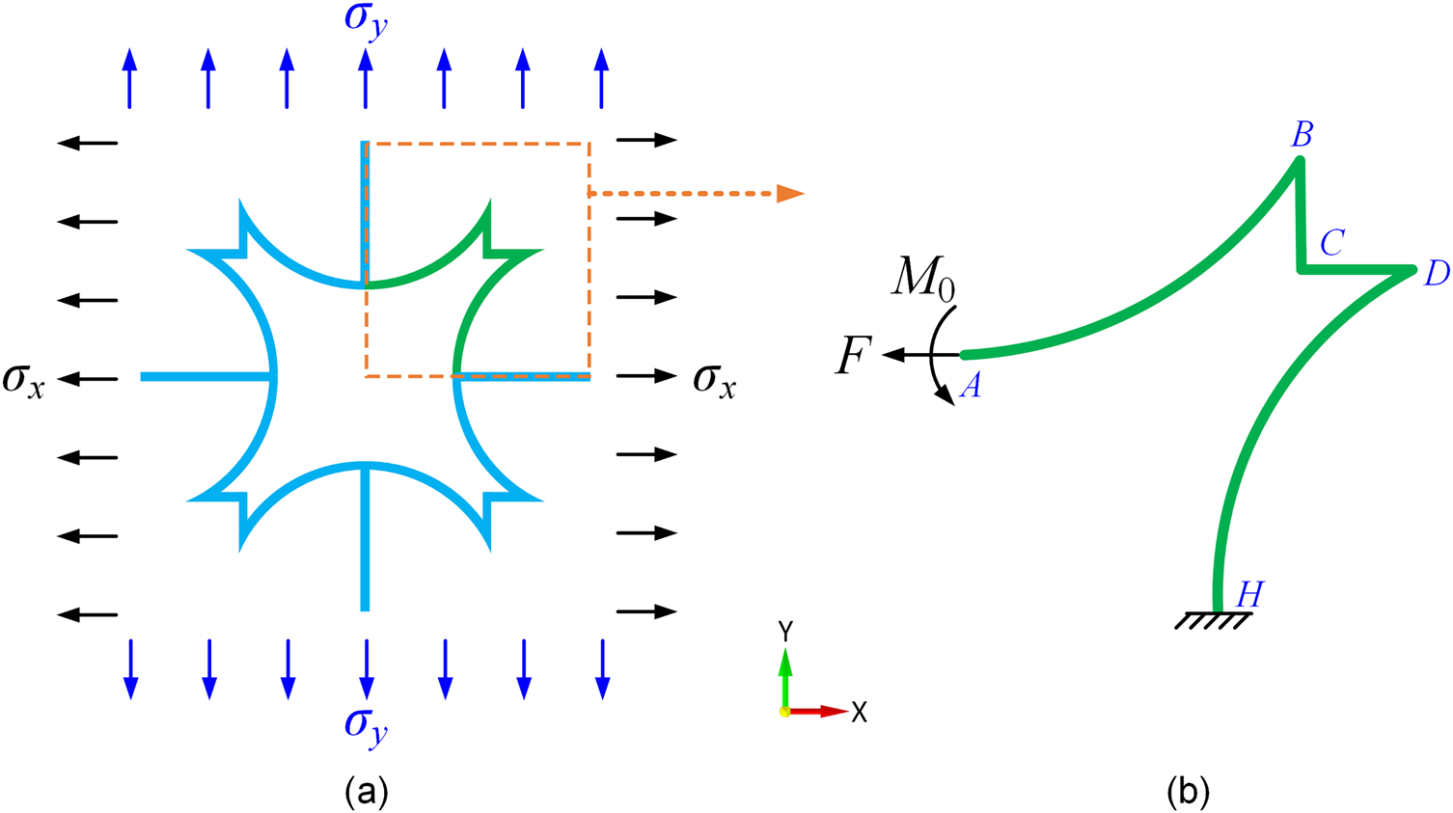
(**a**) The CFAH structure loading conditions; (**b**) the analytical quarter model of the CFAH structure.

Because of the symmetry of the CFAH structure in the *x*-and *y-*directions, it has the same mechanical properties in both directions, as shown in Fig. 2a. Based on Castigliano’s second theorem and Moore’s theorem, the in-plane elastic modulus of the quarter CFAH structure along the *x*-direction is investigated, as shown in Fig. 2b. In this paper, the deformations caused by the straight and curved rods are considered as the main contribution to the in-plane uniaxial tensile of the CFAH structure.

As shown in Fig. 2b, the bending moment equations on the beam sections of *AB*, *BC*, *CD* and *DH* are *M*_1_, *M*_2_, *M*_3_ and *M*_4_, respectively. Then the bending moment equations on these five beams are:

5 $$M_{1} = - F\left( {R - R{\text{cos}}\varphi } \right) + M_{0} {\kern 1pt} {\kern 1pt} {\kern 1pt} {\kern 1pt} {\kern 1pt} {\kern 1pt} {\kern 1pt} {\kern 1pt} {\kern 1pt} {\kern 1pt} \varphi \in \left( {0,\theta } \right)$$

6 $$M_{2} = - F\left( {R - R{\text{cos}}\theta } \right) + Fx + M_{0} {\kern 1pt} {\kern 1pt} {\kern 1pt} {\kern 1pt} {\kern 1pt} {\kern 1pt} {\kern 1pt} {\kern 1pt} {\kern 1pt} {\kern 1pt} x \in \left( {0,b} \right)$$

7 $$M_{3} = - F\left( {R - R{\text{cos}}\varphi } \right) + Fb + M_{0} {\kern 1pt} {\kern 1pt} {\kern 1pt} {\kern 1pt} {\kern 1pt} {\kern 1pt} {\kern 1pt} {\kern 1pt} {\kern 1pt} {\kern 1pt} x \in \left( {0,b} \right)$$

8 $$M_{4} = - F\left( {R - R{\text{cos}}\theta } \right) + Fb + F + \left[ {R{\text{sin}}\theta - R{\text{sin}}\left( {\theta - \varphi } \right)} \right] + M_{0} {\kern 1pt} {\kern 1pt} {\kern 1pt} {\kern 1pt} {\kern 1pt} {\kern 1pt} {\kern 1pt} {\kern 1pt} {\kern 1pt} {\kern 1pt} \varphi \in \left( {0,\theta } \right)$$

The bending moment *M*_0_ in the beam section can be obtained by using the regular equation of force method:

9 $$M_{0}^{CFAH} = - \frac{{F\left( \begin{gathered} - 4R^{2} \theta + 2R^{2} {\text{sin}}\theta + 3b^{2} - 4Rb + 4Rb{\text{cos}}\theta \hfill \\ + 2R^{2} \theta {\text{cos}}\theta + 2Rb\theta + 2R^{2} \theta {\text{sin}}\theta - 2R^{2} + 2R^{2} {\text{cos}}\theta \hfill \\ \end{gathered} \right)}}{{4\left( {R\theta + b} \right)}}$$

The deformation of each rod can be obtained according to Moore's theorem, and Δ*x* and Δ*y* can be obtained by adding them together:

10 $$\Delta_{x}^{CFAH} = \Delta_{xAB} + \Delta_{xBC} + \Delta_{xCD} + \Delta_{xDH}$$

11 $$\Delta_{y}^{CFAH} = \Delta_{yAB} + \Delta_{yBC} + \Delta_{yCD} + \Delta_{yDH}$$

The deformations Δ_*xAB*_, Δ_*xBC*_, Δ_*xCD*_, Δ_*xDH*_, Δ_*yAB*_, Δ_*yBC*_, Δ_*yCD*_, Δ_*yDH*_ for each rod in Eqs. (10) and (11) can be found in supplementary material.

According to the definition of Poisson's ratio, *υ* is:

12 $$\upsilon^{CFAH} = - \frac{{\varepsilon_{y} }}{{\varepsilon_{x} }}$$

The equivalent Young’s modulus *E* of this structural unit along the *x*-direction can be obtained from the ratio of stress to strain, as follows:

13 $$E^{CFAH} = \frac{\sigma }{{\varepsilon_{x} }}$$

Equations (12) and (13) in *υ*^*CFAH*^ and *E*^*CFAH*^ are listed in the supplementary material.

### Validation

**Figure 3:**
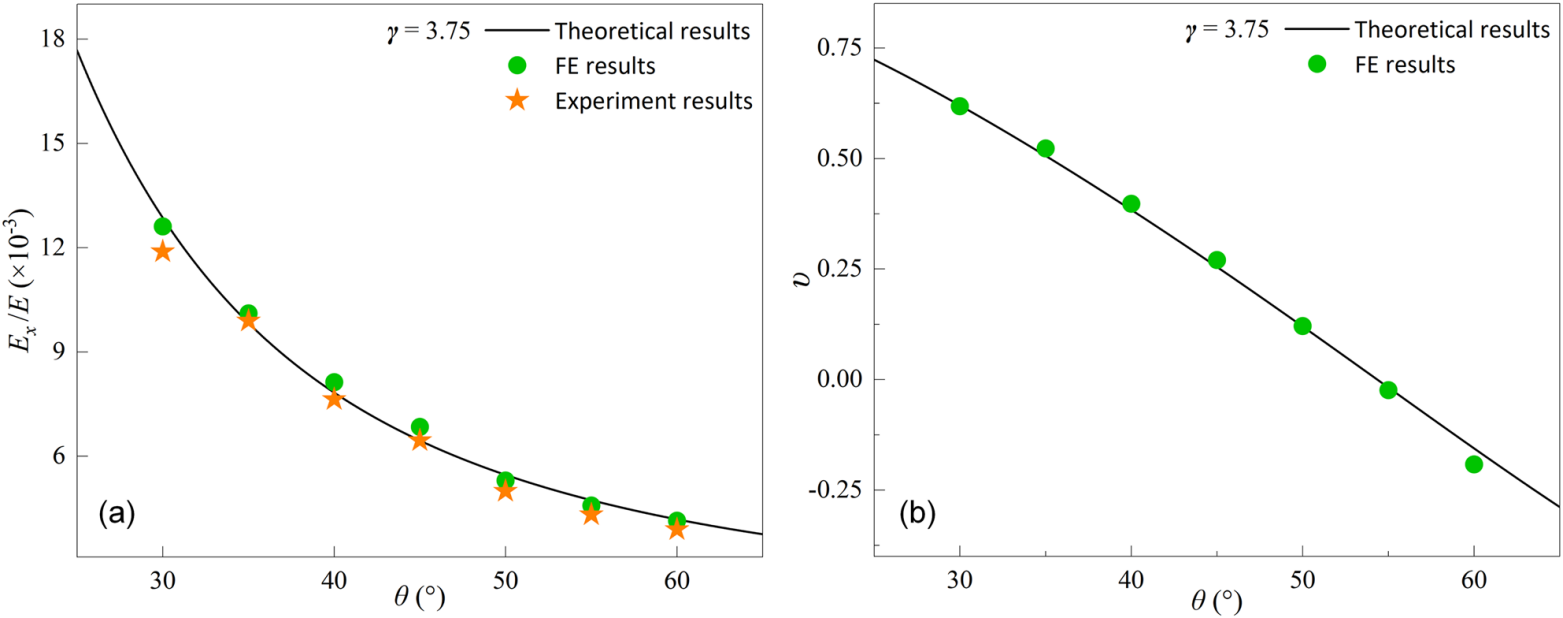
(**a**) Comparison of theoretical, FE and experimental results for normalized effective Young's modulus (*E*_*x*_/*E*); (**b**) Comparison between the theoretical and FE results of Poisson’s ratio (*υ*).

Theoretical results, experimental results, and FE results for normalized effective Young's modulus with different parameters *θ* are compared to verify the theoretical model, as shown in Fig. 3a. The curves show that the theoretical analysis results are in good agreement with both FE results and experimental results. In Fig. 3a, the average error of the three result values for each sample remains within 7%, indicating good agreement among the three results. The maximum average error is 6.51% and the minimum average error is 2.2%. Figure 3b shows the validation of the FE results of Poisson's ratio with the theoretical results. The results of the theoretical analysis are in good agreement with the FE results as well. It can be seen from Fig. 3a that *E*_*x*_/*E* decreases as *θ* increases. In Fig. 3b, *υ* similarly decreases linearly as *θ* increases and achieves a positive to negative Poisson effect shift.

### Effect of structural parameters on the elastic properties of honeycomb

**Figure 4:**
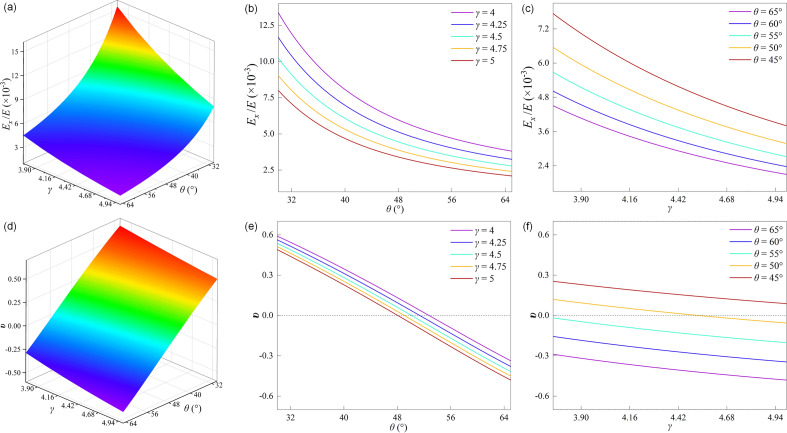
The surface of (**a**) *E*_*x*_/*E* of CFAH with the change of *γ* and *θ*; The curve of *E*_*x*_/*E* of CFAH with the change of (**b**) *θ* when 4 < *γ* < 5; (**c**) *γ* when 45° < *θ* < 65°; The surface of (**d**) *υ* of CFAH with the change of *θ* and *γ*; The curve of *υ* of CFAH with the change of (**e**) *θ* when 4 < *γ* < 5; (**f**) *γ* when 45° < *θ* < 65°;

Figure 4a–c shows the effect of parameters *θ* and *γ* on *E*_*x*_*/E*. Figure 4a shows the trend of *E*_*x*_/*E* when the CFAH structure is stretched along the *x*-direction. *E*_*x*_/*E* decreases with increasing *θ* and *γ*. When 3.75 < *γ* < 5, the growth rate of *E*_*x*_/*E* increases rapidly as *θ* decreases. When 30° < *θ* < 65°, *E*_*x*_/*E* increases slowly as *γ* decreases. When *θ* = 30° and *γ* = 3.75, *E*_*x*_/*E* reaches a maximum value of 15.43 × 10^–3^. When *θ* = 65° and *γ* = 5, *E*_*x*_/*E* reaches a minimum value of 2.09 × 10^–3^. It can be seen from the figure that *E*_*x*_/*E* is more sensitive to *θ*. The effect of design parameters on *E*_*x*_/*E* is shown in Fig. 4b and c. Figure 4b shows that *E*_*x*_/*E* decreases with increasing *θ*, and that it decreases with increasing *γ* when 4 < *γ* < 5. Figure 4c shows that *E*_*x*_/*E* decreases with increasing *γ* and decreases with increasing *θ* when 45° < *θ* < 65°.

Figure 4d–f shows the effect of parameters *θ* and *γ* on *υ*. Figure 4d shows the effect of structural parameters on *υ*. *υ* decreases with increasing *θ* and *γ*, and Poisson's ratio shows a transition from a positive to a negative Poisson's effect. It can be seen from the figure that *υ* increases slowly as *γ* decreases when *θ* is constant. When *γ* is constant, *υ* increases rapidly as *θ* decreases. This means that for the CFAH structure, *γ* has little effect on *υ* and is more sensitive to *θ*. Figure 4e and f show the effect of structural parameters on *υ*. It can be seen from Fig. 4e that *υ* decreases monotonically with increasing *θ* and *γ* when 4 < *γ* < 5. The figure shows that *υ* changes from a positive Poisson effect to a negative Poisson effect as *θ* increases. As shown in Fig. 4f, when 45° < *θ* < 65°, *υ* decreases linearly with increasing *γ* while decreasing with increasing *θ*. When *θ* = 50°, *υ* changes from positive Poisson to negative Poisson effect as *γ* increases. When *θ* = 55°, 60° and 65°, *υ* maintains the negative Poisson effect as *γ* increases.

As can be seen in Fig. 4, for the CFAH structure, the auxiliary effect is closely related to the arc length and curvature of the curved rod. When both *γ* and *θ* increase, the arc length of the curved rod increases, and the unit cell deformation is more significant when subjected to vertical load. The strain of the CFAH structure increases in the *x*-direction and similarly in the *y*-direction, which leads to a more significant auxiliary effect and also to a decrease in* E*_*x*_/*E*. When 3.75 < *γ* < 5 and 45° < *θ* < 65°, the increase of *θ* leads to the growth of the arc length of the curved rod while the curvature remains unchanged, so the effect of the parameter *θ* on *υ* is more significant. However, when *γ* increases, although it also increases the arc length of the curved rod, it decreases the curvature, so the effect on *υ* is lower than the effect of the parameter *θ*.

### Comparison of elastic properties of ISSH structure and CFAH structure

**Figure 5:**
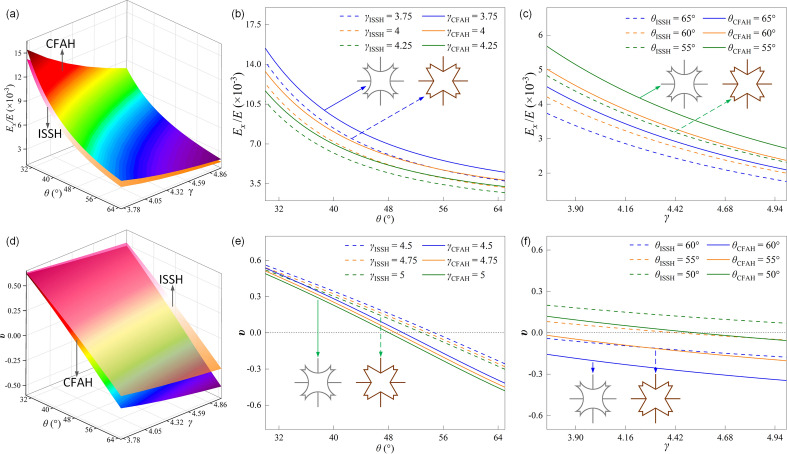
Comparison of the variation of (**a**), (**b**) and (**c**) *E*_*x*_/*E*; (**d**), (**e**) and (**f**) *υ*.

The Young's modulus and Poisson’s ratio of the ISSH structure can be solved by the above theoretical method, and the detailed equations are given in supplementary material. Figure 5 shows the trends of *E*_*x*_/*E* and *υ* with *θ* and *γ* for both structures. It is clear from Fig. 5a that the trend of* E*_*x*_/*E* changes for both structures is the same, and both increase with decreasing *θ* and *γ*. Meanwhile, the *E*_*x*_/*E* of the CFAH structure has been larger than the *E*_*x*_/*E* of the ISSH structure, showing a better load-bearing capacity. Figure 5b illustrates that the *E*_*x*_/*E* of the ISSH and CFAH structures decreases with increasing parameter *θ*. When *γ* = 3.75, 4 and 4.25, the *E*_*x*_/*E* of the CFAH structure is larger than that of the ISSH structure, and the *E*_*x*_/*E* is improved by about 14.81%, 14.91%, and 14.96%, respectively. The *E*_*x*_/*E* decreases with increasing *γ*. Figure 5c shows the decrease of *E*_*x*_/*E* with increasing parameter *γ* for the ISSH and CFAH structures. When *θ* = 55°, 60° and 65°, the *E*_*x*_/*E* of the CFAH structure is also larger than that of the ISSH structure, and the *E*_*x*_/*E* is improved by about 17.62%, 18.89%, and 19.94%, respectively. The *E*_*x*_/*E* decreases with increasing *θ*.

From Fig. 5d it is shown that the *υ* of both structures increases as *θ* and *γ* decrease. Compared to the ISSH structure, the CFAH structure shows more auxiliary effects. When *θ* = 30° or *γ* = 3.75, the difference of Poisson effect between the two structures is not significant, but the difference gradually becomes larger with the increase of *θ* and *γ*. Figure 5e and f show the decrease of *υ* with increasing *θ* and *γ* for the ISSH structure and the CFAH structure. The Poisson's ratio of the CFAH structure is smaller than that of the ISSH structure, regardless of the variation of the structural parameters. The auxiliary effect of the CFAH structure is more significant, for example, when *θ* = 52° and *γ* = 4.5, the Poisson’s ratio for the CFAH structure is − 0.054, while the Poisson's ratio for the ISSH structure is 0.066.

**Figure 6:**
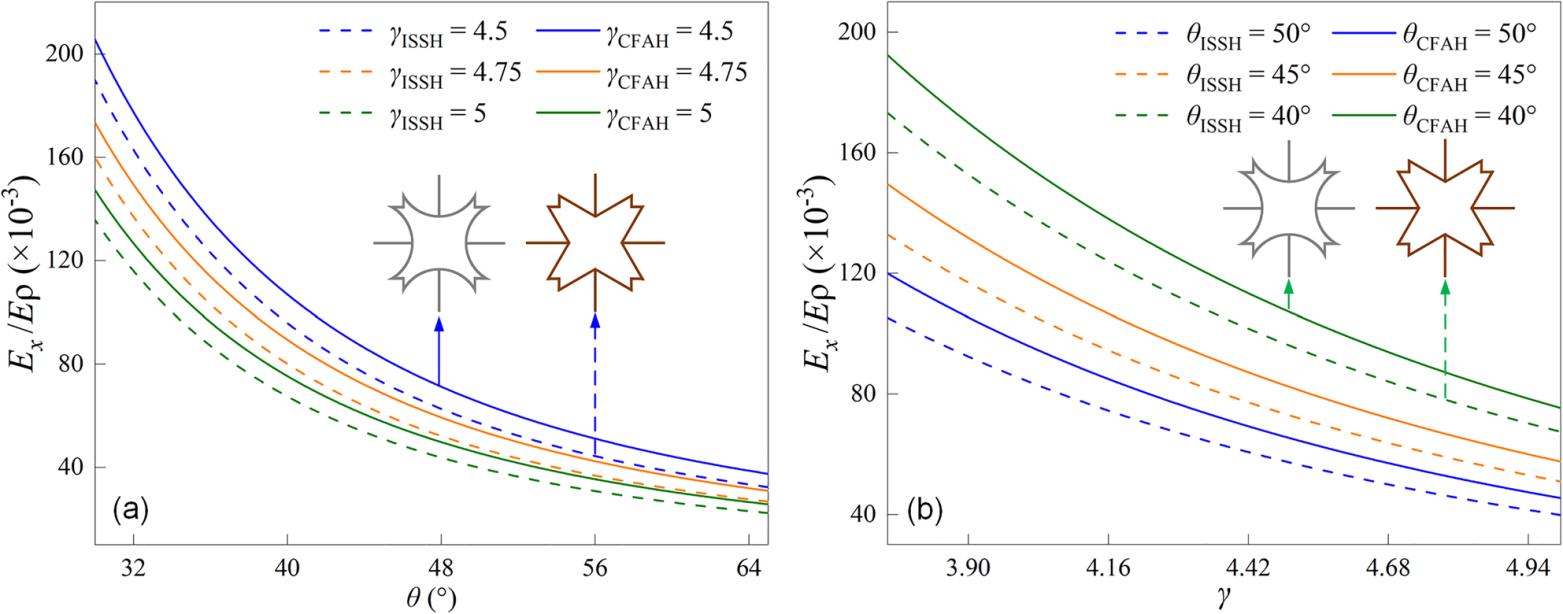
Comparison of the normalized effective specific stiffness (*E*_*x*_/*Eρ*) of the two structures in (**a**) and (**b**).

Figure 6 shows a comparison of the normalized specific stiffness of the two structures. It is obvious from Fig. 6a that *E*_*x*_/*Eρ* decreases with increasing *θ* for both structures. When *γ* = 4.5, 4.75 and 5, the* E*_*x*_/*Eρ* of the CFAH structure is larger than that of the ISSH structure. Compared with the ISSH structure, the *E*_*x*_/*Eρ* of the CFAH structure is improved by about 13.09%, 13.08% and 13.06%, respectively. In Fig. 6b, it is shown that the *E*_*x*_/*Eρ* decreases with increasing* γ* for both structures. The *E*_*x*_/*Eρ* of the CFAH structure is larger than that of the ISSH structure for *θ* = 40°, 45° and 50°. Compared with the ISSH structure, the *E*_*x*_/*Eρ* of the CFAH structure is improved by about 11.45%, 12.89% and 14.12%, respectively.

Figures 5 and 6 clearly show the superior mechanical properties of the CFAH structure. At the same time, since the CFAH structure is designed from a straight rod to a curved rod, there is little difference in the manufacturing process and manufacturing time from the ISSH structure. Both structures are 3D printed, with no change in the fabrication process, bringing no increase in the difficulty of the fabrication process. There is no additional process difficulty for commercial mold production. Fig. S2 in the supplementary material shows the relative densities of the two structures. As can be seen from the figure, the material required to fabricate the CFAH structure is slightly higher than that of the ISSH structure, but the mechanical properties of the CFAH structure are significantly improved. For example, when *θ* is 65°, the material required to make the CAFH structure is only about 3.32% more than the ISSH structure. However, the normalized Young's modulus increased by 19.94%. At the same time, the difference in consumed material decreases as *θ* becomes decreases. This shows the CFAH structure to be more cost-effectiveness and highlights its potential for practical engineering applications.

## Materials and methods

**Figure 7:**
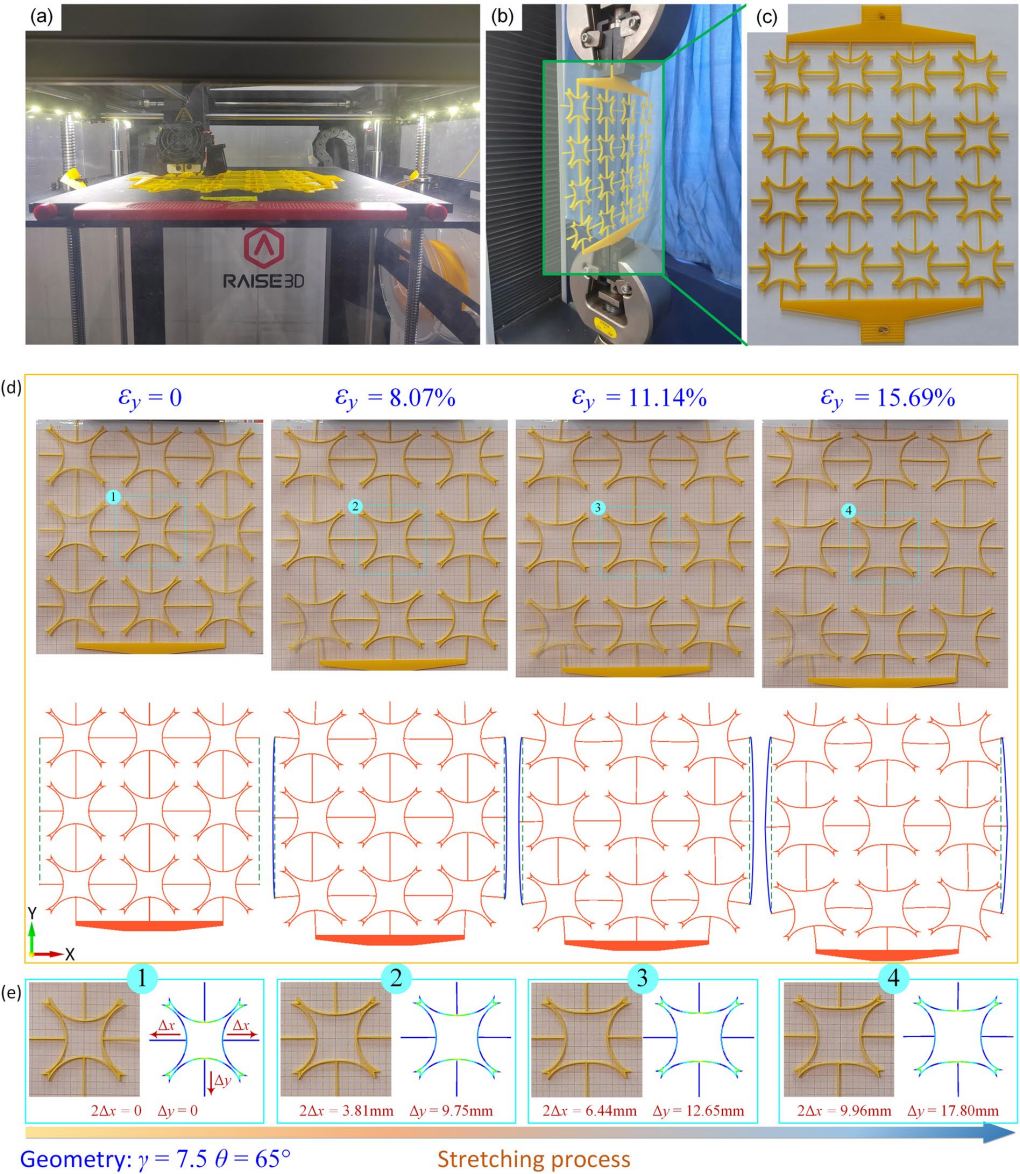
(**a**) the process of 3D Printing; (**b**) uniaxially tensile test; (**c**) CFAH samples; (**d**) The deformation process of CFAH structure sample; (**e**) stress distribution.

All materials and structure samples used in the experiments are fabricated by the Fused Deposition Modeling (FDM) 3D printing method, as shown in Fig. 7a. Young's modulus of the material is determined by tensile testing^39^. Polylactic acid (PLA) has been selected as the process material for FDM 3D printing production. The experimental samples are fabricated using the RAISE 3D Pro2 Plus 3D printing device. The tensile samples are fabricated with the GB/T1040.4-2006/ISO527-4:1997 standard (Plastics-Determination of tensile properties-Part4: Test conditions for isotropic and orthotropic fiber-reinforced plastic composites). In previous work, the Young’s modulus of the PLA material is measured to be 3230 MPa^39^. The Poisson's ratio is 0.35. The mechanical properties of the materials derived from the tests in this paper are consistent with the results of similar works reported by previous authors^45–48^.

In this paper, eight sets of experimental samples are produced by FDM 3D printing technology. Among them, the first seven groups of samples have the same parameter *γ*, and the parameters *θ* are 60°, 55°, 50°, 45°, 40°, 35°, and 30°, respectively. The structural parameters of the last set of samples are *γ* = 7.5 and *θ* = 65°. The quasi-static compression tests of CFAH structures are carried out by 100 kN standard universal testing machine WDW-100. Figure 7b and c show that uniaxial tensile tests with a loading speed of 2 mm/ min are performed on the CFAH samples.

The numerical simulations are performed to evaluate and analyze the CFAH structure model using the commercial software HyperWorks (version 2017, Altair Engineering, Inc.). In the finite element simulation, reasonable boundary conditions are set according to the specific situation during the tensile experiment. The six degrees of freedom at one end of the model are constrained and the load is applied at the other end. To improve the computational accuracy and reduce the computational time, we used a suitable meshing method for the CFAH structure. Due to the regular shape of the CFAH structure, a hexahedral mesh is chosen for the delineation. The finite element mesh consists of 166,560 elements comprising a total of 234,906 nodes.

In Fig. 7, the deformation process and stress distribution during the tensile process of the CFAH experiment are shown. The experimental design is the same as that of the FE simulation. From Fig. 7d, it can be seen that the negative Poisson effect is gradually significant as the strain increases when the sample is tensile in the *y*-direction. Figure 7e shows the deformation and stress distribution of the unit cell at the center of the CFAH structure. It can be seen from the figure that the experimental and FE deformations are consistent. The unit cell stress is mainly distributed in the center of the curved rod and near the re-entrant angle.

## Conclusion

In this paper, the ISSH structure is improved and a novel concave four-arc honeycomb (CFAH) structure is proposed with curved rods instead of straight rods. Meaningfully, the CFAH structure shows enhanced normalized effective specific stiffness and auxiliary effects. The in-plane elastic properties of the CFAH structure are solved using Castigliano's second theorem and Moore's theorem. The theory is validated by experimental and FE methods, the three results are in good agreement. From this present work, the following conclusions can be drawn:

1. The mechanical properties of the CFAH structure are closely related to the curved rod arc length and curvature. According to the parametric study, both *E*_*x*_/*E* and *υ* decrease with increasing *θ* and *γ*. When *θ* increases, the arc length of the curved rod increases, and the curvature stays the same. However, when *γ* increases, the arc length increases and the curvature becomes smaller. Therefore, *E*_*x*_/*E* and *υ* are more sensitive to the parameter *θ* compared to the parameter *γ*.
2. The CFAH structure has enhanced *E*_*x*_/*Eρ* and *E*_*x*_/*E* compared to ISSH. The normalized effective specific stiffness is improved by about 12.95% and normalized effective Young's modulus is improved by about 16.86%. Meanwhile, the CFAH structure always has a smaller *υ* than the ISSH structure. Furthermore, this suggests that the CFAH structure has more significantly auxiliary effects.

This paper provides guidance for the study of star-shaped honeycomb structures, revealing that CFAH structures have enhanced normalized effective specific stiffness and auxiliary effects. This enhanced mechanical property is what makes CFAH structures show good promise for aerospace, construction, and other applications.

### Supplementary Information

Supplementary Information.

## Data Availability

The data that support the findings of this study are available on request from the corresponding author.
