# Supplementary material for: A concave four-arc honeycomb with enhanced stiffness and desirable negative Poisson’s effect
Source: Sci Rep. 2023 Nov 30;13:21144. doi: 10.1038/s41598-023-48570-y (PMC10689748; doi:10.1038/s41598-023-48570-y)
Supplement: Supplementary file 1 — Supplementary Information. [file 41598_2023_48570_MOESM1_ESM.docx]

SUPPLEMENTARY MATERIAL

**A concave four-arc honeycomb with enhanced stiffness and desirable negative Poisson's effect**

Ning Feng^1,2,3*^, Yuanhao Tie^4^, Ronghui Guo^3^, Qingwen Yuan^2^, Fengling Xue^2^, Cheng Li^1^, Liwen Lv^5^, Weibo Xie^1*^

^1^School of Intelligent Manufacturing and Transportation, Chongqing Vocational Institute of Engineering, Chongqing 402260, China

^2^Henan Province Engineering Research Center of Ultrasonic Technology Application, Pingdingshan University, Pingdingshan 467000, China

^3^College of Mechanical and Electrical Engineering, Xinjiang Agricultural University, Urumchi, 830052, China

^4^School of Mechanical and Automotive Engineering, Guangxi University of Science and Technology, Liuzhou 545006, China

^5^School of Resources and Security, Chongqing Vocational Institute of Engineering, Chongqing 402260, China

*Corresponding author: [fengning@pdsu.edu.cn](mailto:fengning@pdsu.edu.cn) and [xieweibo0907@163.com](mailto:xieweibo0907@163.com)

**Supporting information 1. Theoretical analysis of CFAH structures**

Deformation of each rod in the *x*-direction Δ*_xAB_,* Δ*_xBC_,* Δ*_xCD_,* Δ*_xDH_*:

$$\Delta_{xAB}^{CFAH}=\frac{R^{2}(3FR\theta-4FR\sin\theta+FR\cos\theta\sin\theta-2M_{0}\theta+2M_{0}\sin\theta)}{2EI}$$

$$\begin{matrix} \Delta_{xBC}^{CFAH}= \frac{Fb^{3}}{3EI}+\frac{\left[-F\left(R-R\cos\theta\right)+F\left(-R+R\cos\theta\right)+M_{0}\right]b^{2}}{2EI} \\ +\frac{\left[-F\left(R-R\cos\theta\right)+M_{0}\right]\left(-R+R\cos\theta\right)b}{EI} \end{matrix}$$

$$\Delta_{xCD}^{CFAH}=\frac{\left[-F\left(R-R\cos\theta\right)+Fb+M_{0}\right]\left[-\left(R-R\cos\theta\right)+b\right]b}{EI}$$

$$\Delta_{xDH}^{CFAH}=\frac{R\left(\begin{matrix} -4RFb+4R^{2}F-8FR^{2}\cos\theta-4FR^{2}\sin\theta-2RM_{0}+3FR^{2}\cos\theta\sin\theta\\ +4bFR\cos\theta\theta+4bFR\cos\theta+2RM_{0}\cos\theta+4FR^{2}\cos^{2}\theta\\ +5FR^{2}\theta+2Fb^{2}\theta-4bFR\theta-4FR^{2}\sin\theta\theta-4FFR^{2}\cos\theta\theta+2RM_{0}\cos\theta\theta\\ +2RM_{0}\sin\theta\theta-2RM_{0}\theta+2bM_{0}\theta+4FR^{2}\cos\theta\sin\theta\theta+4FR\sin\theta b\theta\end{matrix}\right)}{2EI}$$

The strain of the CFAH structure in the *x*-direction is:

$$\varepsilon_{x}^{CFAH}=\frac{2\Delta_{x}^{CFAH}}{L_{x}}=\frac{2\left(\Delta_{xAB}+\Delta_{xBC}+\Delta_{xCD}+\Delta_{xDH}\right)}{L_{x}}$$

Deformation of each rod in the *y*-direction Δ*_yAB_,* Δ*_yBC_,* Δ*_yCD_,* Δ*_yDH_*:

$$\Delta_{yAB}^{CFAH}=\frac{R^{2}\left(-2FR\cos\theta+FR\cos^{2}\theta+2M_{0}\cos\theta+FR-2M_{0}\right)}{2EI}$$

$$\Delta_{yBC}^{CFAH}=-\frac{F\sin\theta Rb^{2}}{2EI}-\frac{\left[-F\left(R-R\cos\theta\right)+M_{0}\right]\sin\theta Rb}{EI}$$

$$\Delta_{yCD}^{CFAH}=\frac{\left[F\left(R-R\cos\theta\right)-Fb-M_{0}\right]b^{2}}{2EI}-\frac{\left[-F\left(R-R\cos\theta\right)+Fb+M_{0}\right]\sin\theta Rb}{EI}$$

$$\Delta_{yDH}^{CFAH}=-\frac{R\left(\begin{matrix} -2FRb+FR^{2}-2FR^{2}\cos\theta+2M_{0}a\theta+4FR^{2}\cos\theta\sin\theta\theta\\ +2FR\cos b-2FR\sin\theta Rb+2Fb^{2}\theta+FR^{2}\cos^{2}\theta-2FR^{2}\sin^{2}\theta\\ -2MR\sin\theta-2FRb\theta-2FR^{2}\theta\sin\theta-2FR^{2}\theta\cos\theta+2FR^{2}\theta\cos^{2}\theta\\ +2FR^{2}\theta\sin^{2}\theta+2MR\theta\sin\theta+2MR\theta\cos\theta+4FRb\theta\cos\theta+4FRb\theta\sin\theta\end{matrix}\right)}{2EI}$$

The strain of the CFAH structure in the *y*-direction is:

$$\varepsilon_{y}^{CFAH}=\frac{2\Delta_{y}^{CFAH}}{L_{y}}=\frac{2\left(\Delta_{yAB}+\Delta_{yBC}+\Delta_{yCD}+\Delta_{yDH}\right)}{L_{y}}$$

According to the definition of Poisson's ratio, *υ^CFAH^* is:

$$\upsilon^{CFAH}=-\frac{\varepsilon_{y}^{CFAH}}{\varepsilon_{x}^{CFAH}}=-\frac{\Delta_{y}^{CFAH}L_{x}}{\Delta_{x}^{CFAH}L_{y}}$$

The equivalent Young's modulus *E^CFAH^* of this structural unit along the *x*-direction can be obtained from the ratio of stress to strain, as follow:

$$E^{CFAH}=\frac{F}{\varepsilon_{x}^{CFAH}t^{2}}$$

**Supporting information 2. Theoretical analysis of ISSH structures**

**
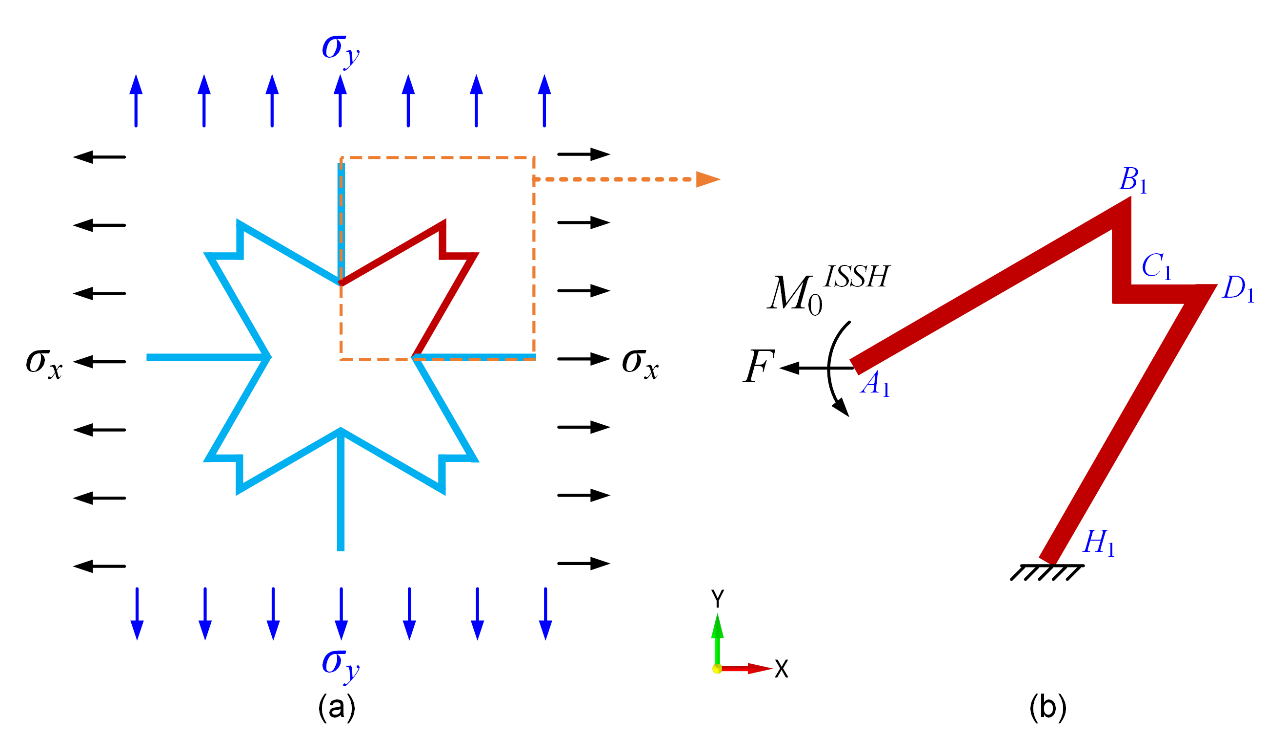
**

**Figure S1.** (a) The ISSH structure loading conditions; (b) the analytical quarter model of the ISSH structure.

As shown in Fig. S1(a) and (b), the bending moment equations on the beam sections of *A*_1_*B*_1_, *B*_1_*C*_1_, *C*_1_*D*_1_ and *D*_1_*H*_1_ are *M*_5_, *M*_6_, *M*_7_ and *M*_8_, respectively. Then the bending moment equations on these four beams are:

$$M_{5}\left(x\right)=-F\cos\theta\cdot x+M_{0}^{ISSH} x\in\left(0,a\right)$$

$$M_{6}\left(x\right)=-F\cos\theta\cdot a+Fx+M_{0}^{ISSH} x\in\left(0,b\right)$$

$$M_{7}\left(x\right)=-F\cos\theta\cdot a+Fb+M_{0}^{ISSH} x\in\left(0,b\right)$$

$$M_{8}\left(x\right)=-F\cos\theta\cdot a+Fb+F\sin\theta\cdot x+M_{0}^{ISSH} x\in\left(0,a\right)$$

The bending moment *M*_0_ in the beam section can be obtained by using the regular equation of force method:

$$M_{0}^{ISSH}=-\frac{F\left(-3\cos\theta a^{2}+3b^{2}-4\cos\theta ab+\sin\theta a^{2}+2ab\right)}{4\left(a+b\right)}$$

The deformation of each rod can be obtained according to Moore's theorem, and Δ*x* and Δ*y* can be obtained by adding them together:

$$\Delta_{x}^{ISSH}=\Delta_{xA_{1}B_{1}}+\Delta_{xB_{1}C_{1}}+\Delta_{xC_{1}D_{1}}+\Delta_{xD_{1}H_{1}}$$

$$\Delta_{y}^{ISSH}=\Delta_{yA_{1}B_{1}}+\Delta_{yB_{1}C_{1}}+\Delta_{yC_{1}D_{1}}+\Delta_{yD_{1}H_{1}}$$

Deformation of each rod in the x-direction:

$$\Delta_{xA_{1}B_{1}}=\frac{a^{2}\cos\theta\left(3M_{0}^{ISSH}-2Fa\cos\theta\right)}{6EI}$$

$$\Delta_{xB_{1}C_{1}}=\frac{b\left(3M_{0}^{ISSH}b+2Fb+6Fa\cos\theta\right)-6M_{0}^{ISSH}a\cos\theta-6Fab\cos\theta}{6EI}$$

$$\Delta_{xC_{1}D_{1}}=\frac{b\left(b-a\cos\theta\right)\left(M_{0}^{ISSH}+Fb-Fa\cos\theta\right)}{EI}$$

$$\begin{matrix} \Delta_{xD_{1}H_{1}}=\frac{a^{2}\sin\theta\left(M_{0}^{ISSH}+Fb-Fa\cos\theta\right)}{2EI}+\frac{F\sin\theta\left(b-a\cos\theta\right)}{2EI} \\ +\frac{a\left(b-a\cos\theta\right)\left(M_{0}^{ISSH}+Fb-Fa\cos\theta\right)}{EI}+\frac{Fa^{2}\sin^{2}\theta}{3EI} \end{matrix}$$

Deformation of each rod in the *y*-direction:

$$\Delta_{yA_{1}B_{1}}=\frac{a^{2}\left(3M_{0}^{ISSH}\sin\theta-2Fa\sin\theta\cos\theta\right)}{6EI}$$

$$\Delta_{yB_{1}C_{1}}=\frac{ab\sin\theta\left(2M_{0}^{ISSH}+Fb-2Fa\cos\theta\right)}{2EI}$$

$$\Delta_{yC_{1}D_{1}}=\frac{b\left(b+2a\sin\theta\right)\left(M_{0}^{ISSH}+Fb-Fa\cos\theta\right)}{2EI}$$

$$\Delta_{yD_{1}H_{1}}=\frac{-\left(\begin{matrix} 3Fa^{3}-3M_{0}^{ISSH}a^{2}\cos\theta+6M_{0}^{ISSH}a^{2}\sin\theta+6M_{0}^{ISSH}ab+6Fab^{2} \\ -8Fa^{3}\cos\theta\sin\theta-9Fa^{2}b\cos\theta+9Fa^{2}b\sin\theta\end{matrix}\right)}{6EI}$$

The strain of the ISSH structure in the *x*-direction, as follows:

$$\varepsilon_{x}^{ISSH}=\frac{2\Delta_{x}^{ISSH}}{L_{x}}=\frac{2\left(\Delta_{xA_{1}B_{1}}+\Delta_{xB_{1}C_{1}}+\Delta_{xC_{1}D_{1}}+\Delta_{xD_{1}H_{1}}\right)}{L_{x}}$$

The strain of the ISSH structure in the *y*-direction is:

$$\varepsilon_{y}^{ISSH}=\frac{2\Delta_{y}^{ISSH}}{L_{y}}=\frac{2\left(\Delta_{yA_{1}B_{1}}+\Delta_{yB_{1}C_{1}}+\Delta_{yC_{1}D_{1}}+\Delta_{yD_{1}H_{1}}\right)}{L_{y}}$$

According to the definition of Poisson's ratio, *υ^ISSH^* is:

$$\upsilon^{ISSH}=-\frac{\varepsilon_{y}^{ISSH}}{\varepsilon_{x}^{ISSH}}=-\frac{\Delta_{y}^{ISSH}L_{x}}{\Delta_{x}^{ISSH}L_{y}}$$

The equivalent Young's modulus *E^ISSH^* of this structural unit along the *x*-direction can be obtained from the ratio of stress to strain, as follow:

$$E^{ISSH}=\frac{F}{\varepsilon_{x}^{ISSH}t^{2}}$$

**Supporting information 3. Relative density**

Fig. S2 shows a comparison of the relative densities of the CFAH structure and the ISSH structure. It is clear from the figure that the relative densities of the two structures increase linearly as *γ* increases. Meanwhile, the difference in relative density between the two structures gradually decreases as *θ* decreases.

**
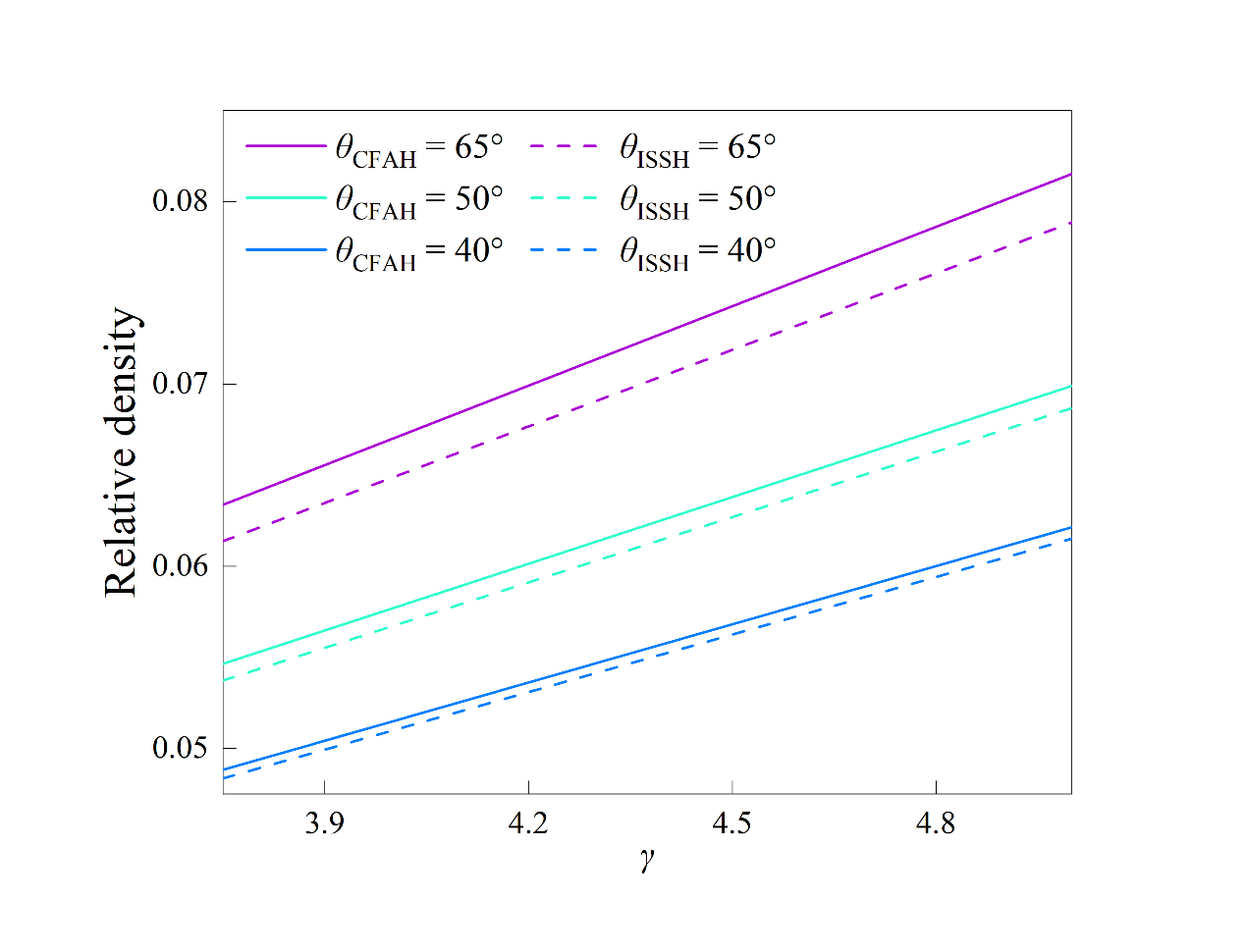
**

**Figure S2.** Relative density.
